# Xyloketal B Exhibits Its Antioxidant Activity through Induction of HO-1 in Vascular Endothelial Cells and Zebrafish

**DOI:** 10.3390/md11020504

**Published:** 2013-02-18

**Authors:** Zhen-Xing Li, Jian-Wen Chen, Feng Yuan, Yun-Ying Huang, Li-Yan Zhao, Jie Li, Huan-Xing Su, Jie Liu, Ji-Yan Pang, Yong-Cheng Lin, Xi-Lin Lu, Zhong Pei, Guan-Lei Wang, Yong-Yuan Guan

**Affiliations:** 1 Department of Pharmacology, Zhongshan School of Medicine, Sun Yat-Sen University, Guangzhou 510080, China; E-Mails: lahm16@yahoo.com.cn (Z.-X.L.); yuanfeng@mail2.sysu.edu.cn (F.Y.); remember917@163.com (Y.-Y.H.); zy201758@126.com (L.-Y.Z.); liujie@mail.sysu.edu.cn (J.L.); guanyy@mail.sysu.edu.cn (Y.-Y.G.); 2 Department of Pharmacology and Toxicology, School of Pharmaceutical Sciences, Sun Yat-Sen University, Guangzhou 510006, China; E-Mail: 573016880@qq.com; 3 Department of Anesthesiology, The Second Affiliated Hospital, Sun Yat-Sen University, Guangzhou 510080, China; E-Mail: mdlijie@sina.com; 4 Key Laboratory of Quality Research in Chinese Medicine, Institute of Chinese Medical Sciences, University of Macau, Macao, China; E-Mail: huanxingsu@umac.mo; 5 Department of Applied Chemistry, School of Chemistry and Chemical Engineering, Sun Yat-Sen University, Guangzhou 510080, China; E-Mails: cespjy@mail.sysu.edu.cn (J.-Y.P.); ceslyc@mail.sysu.edu.cn (Y.-C.L.); 6 Guangdong Province Key Laboratory of Functional Molecules in Oceanic Microorganism, Sun Yat-Sen University, Bureau of Education, Guangzhou 510080, China; 7 Department of Neurology, The First Affiliated Hospital, Sun Yat-Sen University, Guangzhou 510080, China; E-Mail: goodxilin@163.com

**Keywords:** xyloketal B, apoptosis, reactive oxygen species, HO-1, Nrf-2

## Abstract

We previously reported that a novel marine compound, xyloketal B, has strong antioxidative actions in different models of cardiovascular diseases. Induction of heme oxygenase-1 (HO-1), an important endogenous antioxidant enzyme, has been considered as a potential therapeutic strategy for cardiovascular diseases. We here investigated whether xyloketal B exhibits its antioxidant activity through induction of HO-1. In human umbilical vein endothelial cells (HUVECs), xyloketal B significantly induced HO-1 gene expression and translocation of the nuclear factor-erythroid 2-related factor 2 (Nrf-2) in a concentration- and time-dependent manner. The protection of xyloketal B against angiotensin II-induced apoptosis and reactive oxygen species (ROS) production could be abrogated by the HO-1 specific inhibitor, tin protoporphyrin-IX (SnPP). Consistently, the suppressive effects of xyloketal B on NADPH oxidase activity could be reversed by SnPP in zebrafish embryos. In addition, xyloketal B induced Akt and Erk1/2 phosphorylation in a concentration- and time-dependent manner. Furthermore, PI3K inhibitor LY294002 and Erk1/2 inhibitor U0126 suppressed the induction of HO-1 and translocation of Nrf-2 by xyloketal B, whereas P38 inhibitor SB203580 did not. In conclusion, xyloketal B can induce HO-1 expression via PI3K/Akt/Nrf-2 pathways, and the induction of HO-1 is mainly responsible for the antioxidant and antiapoptotic actions of xyloketal B.

## 1. Introduction

Oxidative stress plays a critical role in the pathogenesis of many cardiovascular diseases, including atherosclerosis, and is therefore an attractive therapeutic target for cardiovascular diseases. Recently, many antioxidant compounds have been developed for the treatment of cardiovascular diseases. For example, ebselen, a seleno-organic compound, has demonstrated its clinical efficacy and has been approved for clinical use in different countries, including Japan and China [1,2]. 

**Figure 1:**
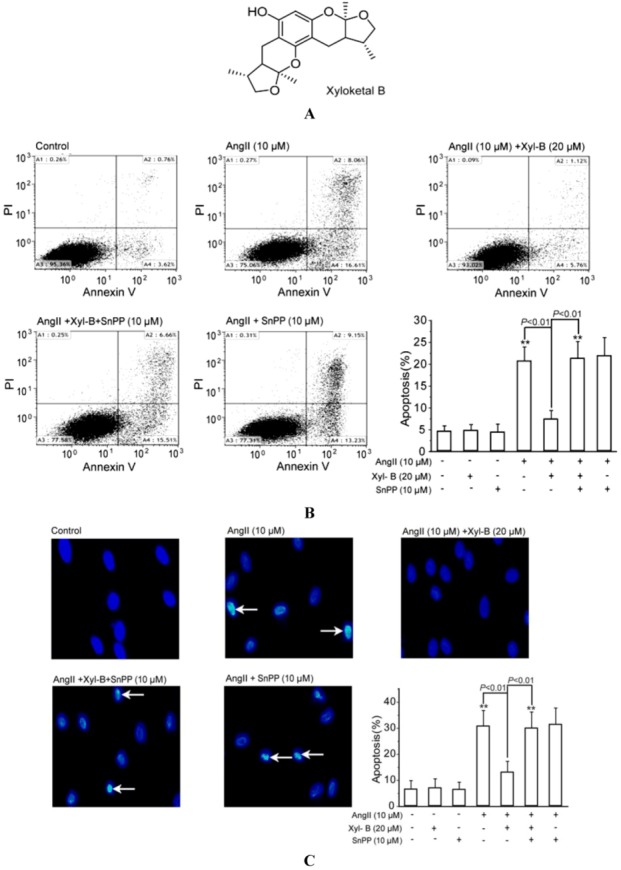
HO-1 contributes to xyloketal B protection against Ang II-induced apoptosis in human umbilical vein endothelial cells (HUVECs). (**A**) The chemical structure of xyloketal B; (**B**) flow cytometric analysis of apoptosis in HUVECs via Annexin V-FITC/PI staining. HUVECs were pretreated with or without 20 μM of xyloketal B for 30 min, in the presence or absence of 10 μM tin protoporphyrin-IX (SnPP), respectively, then followed by exposure to 2 μM AngII for 24 h. The mean values of the percentage of apoptotic cells (A2 + A4) of different groups were given as the mean ± SD and summarized by the bar graph (*n* = 6). (**C**) DAPI staining was employed to detect apoptotic cell death. Cellular pyknosis is indicated with the white arrow. The mean values of the percentage of apoptotic cells were summarized by the bar graph (*n* = 6). ** *p* < 0.01 *vs.* vehicle control.

Xyloketal B is a novel marine compound with a unique chemical structure, isolated from mangrove fungus *Xylaria* sp. (no. 2508) (Figure 1A) [3]. We have demonstrated that xyloketal B can protect against a variety of pathophysiological stimuli, such as oxLDL, oxygen-glucose deprivation (OGD) and MPP+, in different disease models [4,5,6]. Strikingly, the therapeutic efficacy of xyloketal B is comparable to ebselen [6]. Thus, xyloketal B might be a good candidate for further development as an antioxidant medicine in cardiovascular diseases. Based on our previous observations, we believe that the beneficial actions of xyloketal B are largely associated with its antioxidant properties. However, the relatively weak direct scavenging activity of xyloketal B cannot fully explain its potent antioxidant action [6]. In addition to direct antioxidant action (scavenging reactive oxygen species [ROS]/reactive nitrogen species [RNS]), many antioxidant compounds also exert their antioxidant effects by activating the endogenous antioxidant defense system. Interestingly, we found that xyloketal B can also induce some endogenous antioxidant proteins, such as GSH and Bcl-2 [4,5]. Therefore, we hypothesized that xyloketal B may protect against oxidant insults via modulating the endogenous antioxidant system.

Heme oxygenase-1 (HO-1) is one of the critical components in the endogenous antioxidant system of the body. HO-1 is a stress-inducible rate-limiting enzyme in the metabolism of heme, releasing the bioactive molecules, carbon monoxide (CO), biliverdin and iron, that are involved in the defense and repair system of organism against oxidative stress [7]. In vascular and endothelial cells when confronted with oxidative stress, hemodynamic stress and nitric oxide, the induction of the HO-1 gene is mainly regulated by the activation of the transcription factor nuclear factor erythroid 2-related factor 2 (Nrf-2), PI3k/Akt and the MAPK/ERK pathway [8,9,10]. Substantial studies have recognized HO-1 as an important therapeutic target for the treatment of cardiovascular diseases with high oxidative-stress levels, such as hypertension, atherosclerosis, diabetes, obesity and myocardial ischemia-reperfusion injury [11,12,13,14]. In fact, many well-known and commonly used cardiovascular drugs have been reported to modulate HO-1 activity and/or expression [11,15]. 

During the past ten years, zebrafish have become a new popular model in the field of cardiovascular research, particularly for the high-throughput screening. Compared with other vertebrate models, zebrafish embryos develop rapidly, are transparent and small in size [16,17]. Therefore, zebrafish embryos make an ideal model system for *in vivo* studies of biological activities [18]. For example, the activity of NADPH oxidase, a major source of ROS in the cardiovascular system, has been successfully measured *in vivo* in respiratory burst assays in zebrafish embryos [19]. 

In the present study, we investigated the effects of xyloketal B on the HO-1 gene induction and signaling pathways involved in xyloketal B-induced HO-1 expression in human umbilical vein endothelial cells (HUVECs) *in vitro*, as well as its effects on NADPH oxidase activity in zebrafish embryos *in vivo*.

## 2. Results

### 2.1. Involvement of HO-1 in the Protective Effects of Xyloketal B on AngII-Induced Apoptosis in HUVECs

The induction of the HO-1 gene and the subsequent suppressing NADPH oxidase derived superoxide generation are central features of antioxidant therapy against Ang-II induced cardiovascular injury [20,21,22]. Our previous reports have demonstrated the anti-apoptotic and antioxidant effects of xyloketal B in HUVECs [4]. By using the similar culture system, we have shown that xyloketal B has no toxic effects on HUVECs in concentrations up to 160 μM by MTT assay. Accordingly, xyloketal B at the concentration of 20 μM was applied for the following Ang-II (10 μM)-induced HUVEC apoptosis and intracellular ROS production experiments. To test the effects of xyloketal B on Ang-II-induced apoptosis, we performed the Annexin V-FITC/PI binding assay to evaluate phosphatidylserine translocated from the inner surface of the membrane to the outside of the cell, which is specific to the early stage of cell apoptosis. As shown in Figure 1B, 10 μM AngII significantly increased the percentage of apoptotic cells, which was significantly reduced by pretreatment with xyloketal B (20 µM) for 30 min. When the HO-1 specific inhibitor, SnPP (10 μM), was given individually or 30 min prior to the addition of xyloketal B, the protection of xyloketal B against Ang-II-induced apoptosis was fully abrogated by pretreatment with SnPP. SnPP alone did not exhibit either toxic or proliferative effects on HUVECs. In addition, SnPP alone did not affect Ang-II-induced apoptosis. 

DAPI staining was performed to assess morphological changes of the cells and the late events during apoptosis, such as nuclear segmentation and chromatin condensation (Figure 1C). The incubation of HUVECs with AngII (10 μM) could induce these cell apoptotic morphological changes, which were reversed by 20 µM xyloketal B. Also, the HO-1 inhibitor, SnPP, attenuated the protective effects of xyloketal B. The results from both apoptotic assays strongly suggested that HO-1 plays an important role in the anti-apoptotic effects of xyloketal B.

### 2.2. Involvement of HO-1 in the Inhibitory Effect of Xyloketal B on Ang II-Induced ROS Overproduction in HUVECs and PMA-Induced Respiratory Burst of Zebrafish Embryos

**Figure 2:**
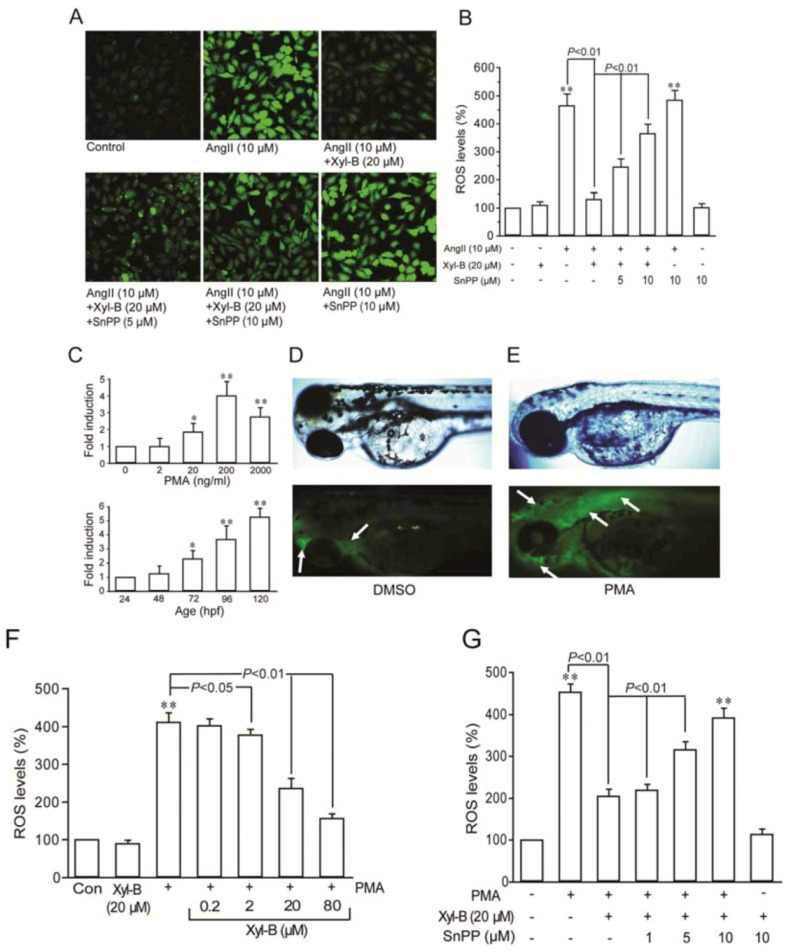
HO-1 contributes to xyloketal B inhibition of reactive oxygen species (ROS) overproduction *in vitro* and *in vivo*. (**A**) The Ang II-induced intracellular ROS production in HUVECs were measured by H_2_DCFDA fluorescence intensity. Representative fluorescent images were chosen from six separating experiments (200×). (**B**) The mean values of DCF fluorescence intensity of different group were normalized to that containing culture media only and given as the mean ± SD. ** *p* < 0.01 *vs.* vehicle control. (**C**) Concentration-dependent (upper panel) and time-dependent (lower panel) development of fold induction of DCF fluorescence in phorbol myristate acetate (PMA)-stimulated respiratory burst in zebrafish embryos. * *p* < 0.05, ** *p* < 0.01 *vs.* vehicle group (upper); * *p* < 0.05, ** *p* < 0.01 *vs.* 24 hpf (hours post fertilization) group (lower). (**D**,**E**) DIC (upper panel) and fluorescence (lower panel) images of whole zebrafish embryos. Embryos were incubated in 1 μg/mL H_2_DCFDA and 0.1% DMSO (**D**) or 200 ng/mL PMA (**E**) for 60 min. (**F**,**G**) The PMA-induced ROS levels in zebrafish embryos were measured after treatment with increasing concentration of xyloketal B (**F**) or treatment with 20 μM xyloketal B in the presence of SnPP at various concentrations (**G**). The mean values summarized in each bar graph represent the average from six separating experiments. ** *p* < 0.01 *vs.* vehicle control.

DCF florescent staining demonstrated that xyloketal B greatly attenuated AngII-induced overproduction of ROS in HUVECs, which is similar to what we found earlier in several *in vitro* oxidative injury models [4,5,6]. When SnPP (5 and 10 μM) was applied, the inhibition of Ang II-induced ROS production by xyloketal B was partially reversed in a concentration-dependent manner (*p* < 0.01, Figure 2A,B). These results supported that xyloketal B protects against AngII-induced endothelial injury through its antioxidant actions, probably through HO-1, an important intrinsic antioxidant enzyme.

The role of HO-1 in the antioxidant action of xyloketal B was further examined in zebrafish embryos *in vivo* [19]. Consistent with the previous report [19], phorbol myristate acetate (PMA) at the concentration range of 2~2000 ng/mL could concentration-dependently induce a rapid increase in ROS in embryos at 72–96 hpf (hours post fertilization), with the maximum stimulation at the concentration of 200 ng/mL (Figure 2C,D). Therefore, PMA at 200 ng/mL was used to stimulate zebrafish embryos from 72 to 96 hpf to produce ROS in the following experiments. Figure 2E,F shows DMSO-treated and PMA-induced ROS production in DCF-stained embryos. In PMA-induced embryos, fluorescence was evident throughout the embryo (white arrow), indicating the successful establishment of the embryo respiratory burst model. As shown in Figure 2F, compared with the control, the relative intracellular ROS levels in xyloketal B-alone-treated embryos was 90% ± 23%, suggesting that xyloketal B alone did not have any effect on the basal ROS level (*p* > 0.05). Compared with the control, the relative ROS levels in PMA-stimulated embryos were 412% ± 66%, and were 409% ± 47%, 378% ± 56%, 237% ± 67% and 157% ± 32% in the presence of xyloketal B at the concentration of 0.2, 2, 20 or 80 μM, respectively. These data demonstrate that preincubation with xyloketal B in the concentration range of 2–80 μM for 30 min could reduce PMA-induced ROS production in a concentration-dependent manner. As shown in Figure 2G, the administration of SnPP prior to xyloketal B concentration-dependently abrogated the inhibitory effects of xyloketal B on PMA-induced ROS production. The results from the *in vivo* model organism were coincident with that from the *in vitro* cell model, confirming that HO-1 induction could be associated with the antioxidant action of xyloketal B.

### 2.3. Xyloketal B Induced HO-1 Expression in HUVECs

**Figure 3:**
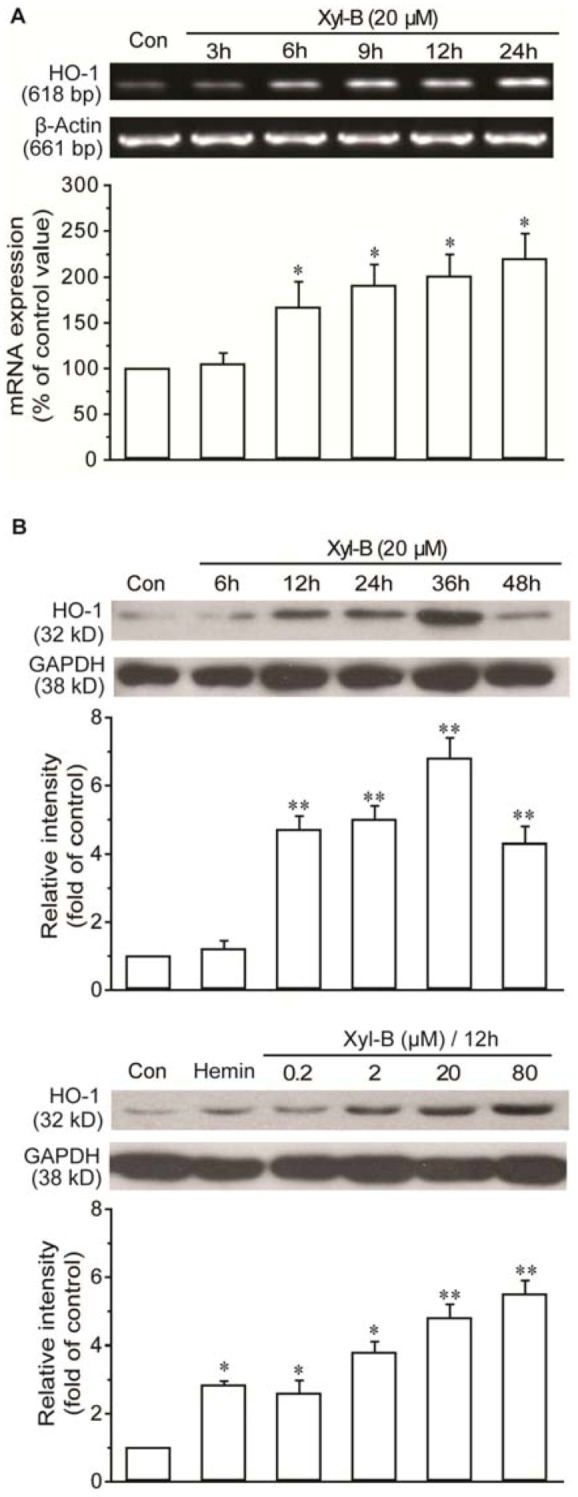
Xyloketal B induced the expression of HO-1. (**A**) Xyloketal B (20 μM) led to a significant increase in HO-1 mRNA expression in HUVECs, with increasing time incubation. Representative results from three separating experiments. (**B**) Western blotting analysis demonstrated that xyloketal B induced HO-1 protein expression in HUVECs by incubation with 20 μM xyloketal B for the indicated time span (upper) or with xyloketal B at increasing concentrations (lower) for 12 h. The HO-1 activator hemin (20 μM) served as a positive control. Each representative blot was chosen from five separating experiments. Results are presented as the mean ± SD. * *p* < 0.05 and ** *p* < 0.01 *vs.* vehicle control.

The cardiovascular protective effect of many drugs is associated with their ability to induce HO-1 gene expression. We then investigated whether xyloketal B directly affected HO-1 expression in HUVECs. As shown in Figure 3A, when incubated with 20 µM xyloketal B for up to 24 h, HO-1 mRNA expression was induced in a time-dependent manner. The incubation of HUVECs with 20 µM xyloketal B for 6 h led to a two-fold induction of HO-1 mRNA when compared with that treated with the vehicle alone. Western blot revealed a similar expression pattern of HO-1 protein following treatment with xyloketal B. The expression level of HO-1 induced by xyloketal B was comparable to hemin, a HO-1 activator. (Figure 3B,C). 

### 2.4. Xyloketal B Induced Activation of Translocation of Nrf-2 and Antioxidant Response Element (ARE) Binding in HUVECs

**Figure 4:**
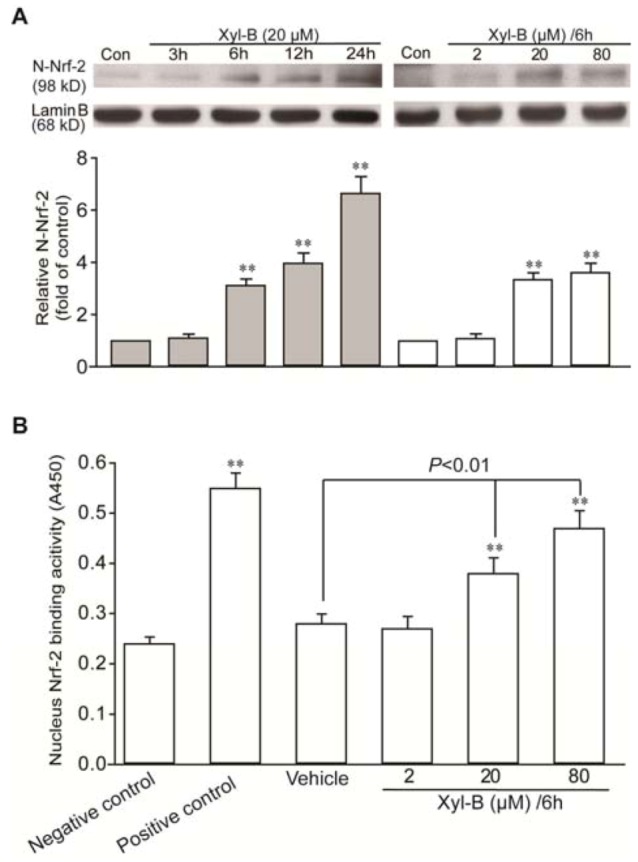
Xyloketal B induced the nuclear translocation of nuclear factor-erythroid 2-related factor 2 (Nrf-2) and binding to antioxidant response element (ARE) in HUVECs. (**A**) Western blotting analysis demonstrated that xyloketal B induced Nrf-2 accumulation in the nuclear extract in HUVECs by incubation with 20 μM xyloketal B for the indicated time span (left) or with xyloketal B at increasing concentrations (right) for 6 h. The representative blot from three separating experiments is shown. ** *p* < 0.01 *vs.* corresponding vehicle control. (**B**) ELISA-based TransAM Nrf-2 assay demonstrated that xyloketal B enhanced the binding of Nrf-2 to its consensus oligonucleotide in the nuclear extract in HUVECs. “Negative control” means without nuclear extract. The positive control is supplied in the TransAM Nrf-2 Kit. Data represent the mean ± SD of four separating experiments. ** *p* < 0.01 *vs.* negative control.

Nrf-2/antioxidant response element (ARE), a transcriptional regulator, is mainly responsible for the induction of HO-1 by many agents [9,23,24]. Nuclear extracts were prepared from HUVECs treated with or without xyloketal B. As shown in Figure 4A, xyloketal B induced Nrf-2 nuclear translocation in a concentration- and time-dependent manner. When cells were treated with xyloketal B at 20 µM for 6 h, xyloketal B led to a 2.3-fold increase in the nuclear accumulation of Nrf-2 protein. 

Similar results were obtained from the ARE-binding assay. Serum-starved HUVECs exhibited endogenous Nrf-2 binding activity to the ARE (Figure 4B). When cells were treated with xyloketal B at various concentrations for 6 h, xyloketal B at 20 and 80 µM enhanced the Nrf-2 DNA-binding activity by 1.4- and 1.7-fold, respectively, compared with the vehicle control. These data suggest that xyloketal B may specifically induce functional HO-1 expression through translocation of Nrf-2 to the nucleus and binding to ARE. 

### 2.5. Effects of Xyloketal B on PI3K/Akt and ERK Signaling in HUVECs

**Figure 5:**
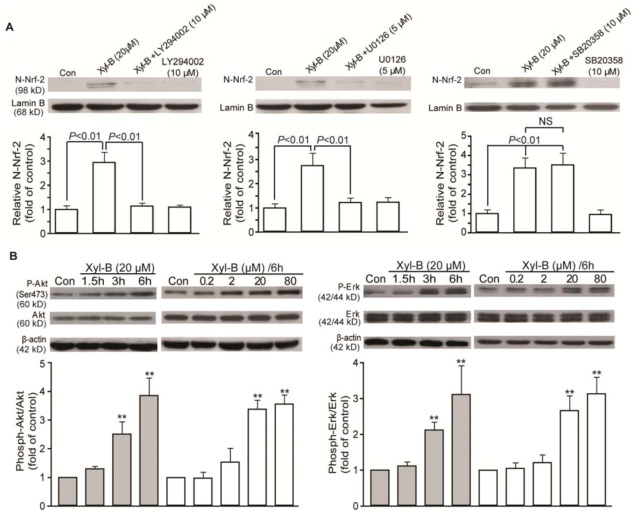
Xyloketal B regulated PI3K/Akt and MAP kinase Erk1/2, upstream signaling of Nrf-2. (**A**) Serum-starved HUVECs were pretreated with the indicated PI3K and MAP kinase inhibitors for 30 min and then incubated with 20 μM xyloketal B for 24 h. Western blotting analysis demonstrated that LY294002 and U0126 abrogated nuclear translocation of Nrf-2 activated by xyloketal B, but not SB203580. (**B**) Xyloketal B induced Akt and Erk1/2 phosphorylation in concentration- and time-dependent manners. HUVECs were treated with an increasing concentration of xyloketal B for 6 h or with 20 μM xyloketal B for the indicated time; then, Western blotting analysis was performed using antibodies specific for phosphorylated Akt (Ser473, P-Akt), phosphorylated Erk1/2 (P-Erk); membranes were stripped and reprobed for the total form of each antibody, respectively. β-actin served as the internal control. P-Akt and P-Erk levels were analyzed and normalized to the vehicle-treated control. Results are presented as the mean ± SD. ** *p* < 0.01 *vs.* corresponding vehicle control.

PI3K/Akt and MAPK signals have been suggested in regulating Nrf-2 translocation and subsequent functional HO-1 induction in response to different stimuli and agents [9,23]. Therefore, a range of inhibitors were applied to examine how these kinases regulate xyloketal B-induced Nrf-2 translocation. As shown in Figure 5A, the xyloketal B-induced nuclear Nrf-2 translocation were inhibited by LY294002 (10 μM), a PI3 kinase inhibitor, and U0126 (5 μM), an Erk1/2 MAPK inhibitor, but not by SB203580 (10 μM), a P38 MAPK inhibitor. LY294002 and U0126 could almost abrogate nuclear Nrf-2 translocation induced by 20 μM xyloketal B. 

We additionally examined whether xyloketal B affected phosphorylated kinases. As shown in Figure 5B, xyloketal B markedly activated Akt phosphorylation (Ser473), phosphor-Erk1 (44 kDa) and phosphor-Erk2 (42 kDa) in both concentration- and time-dependent manners. Xyloketal B at 20 μM strongly induced activation of Akt and Erk 1/2 3 h after treatment and sustained until 6 h after treatment. Parallel blots were performed and probed with antibodies that detected total protein levels of Akt and Erk1/2, indicating that xyloketal B had no effects on total protein expression. These results suggest that xyloketal B-induced HO-1 upregulation mainly depends on activation of Nrf-2 nuclear translocation and involves PI3K/Akt and/or Erk 1/2 MAPK signaling.

## 3. Experimental Section

### 3.1. Chemicals

Xyloketal B was isolated and purified from mangrove fungus *Xylaria* sp. (no. 2508), obtained from the South China Sea, as previously described [3] (Figure 1A), and the purity was >99%. Xyloketal B was dissolved in dimethyl sulfoxide (DMSO) and stored at −20°C until use. The final concentration of DMSO in the culture media was <0.1%.

### 3.2. Zebrafish Husbandry

The wild-type (AB strain) zebrafish were maintained at 28 ± 0.5 °C in a 14 h light/10 h dark cycle in a continuous flow-through system in de-ionized water with a supplement of 10% per day. Fish were fed with live *Artemia nauplii* (3 mL per 10 L water) three times daily, at 8 a.m., 12 p.m. and 5 p.m., respectively, after the flow-through water was suspended. 30 min after feeding, less than 8 zebrafish were put into tanks with a female-male ratio 1:1 to 2:1, separated with a clapboard overnight. The clapboard was removed the next morning, female zebrafish ovulated about 15 min later, and fertilized eggs were obtained when the ovulation was over, then washed and cultured at 28.5 °C. Lethal eggs and unfertilized eggs were eliminated at regular intervals. Embryos between 3 and 4 dpf (days post fertilization) were used in the assay. 

### 3.3. Cell Culture

Human umbilical vein endothelial cells (HUVECs) were isolated and cultured, as we described previously [4]. HUVECs were obtained from human umbilical veins after collagenase type I digestion and cultured in medium 199 (Gibco, USA) containing 20% fetal calf serum (Gibco, USA), penicillin (100 U/mL), streptomycin (100 U/mL) and heparin (50 U/mL), supplemented with L-glutamine (2 mM), sodium pyruvate (1 mM) and endothelial cell growth factor (β-ECGF, 5 ng/mL), at 37 °C in 5% CO_2_ on 1% gelatin-coated culture flasks. Endothelial cells were identified by their morphology, which appears as a “cobblestone” mosaic appearance after reaching confluence and more than 95% of cells presenting Factor VIII-related antigen. Passage 2~5 HUVECs were used for experiments. 

### 3.4. Cell Apoptosis Assay by Annexin V-FITC (Fluorescein Isothiocyanate)/Propidium Iodide (PI) Staining and DAPI Staining

#### 3.4.1. Annexin V-FITC/PI Staining

HUVECs (at a density of 3 × 10^3^/mL) were cultured in 60 mm culture dishes, 2% fetal bovine serum/M199 medium-starved for 12 h and with or without 20 μM xyloketal B for 30 min, in the presence or absence of 10 μM SnPP, respectively, then followed by exposure to 2 μM Ang II for 24 h. According to the manufacturer’s protocol (Sigma, St. Louis, MO, USA), briefly, cells were washed twice with PBS and incubated in 500 µL binding buffer containing 5 µL of Annexin V-FITC and 5 µL of PI in the dark for 5~15 min at room temperature. The stained samples were then analyzed on a FACSort flow cytometer within an hour following the manufacturer’s protocol (Coulter, Hialeah, FL, USA).

#### 3.4.2. DAPI Staining

As described in our previous study [4], HUVECs were treated as above and then fixed in 4% paraformaldehyde for 15 min, incubated with 2.5 μg/mL of DAPI (Molecular Probes, USA) for a further 30 min at room temperature. Photographed nuclei were visualized using laser confocal microscope (200×). Cells with condensed chromatin or shrunken, irregular or fragmented nuclei were considered apoptotic. Apoptotic values were calculated as the percentage of apoptotic cells relative to the total number of cells in each random chosen microscopic field (>200 cells).

### 3.5. ROS Measurement

Reactive oxygen species (ROS) in HUVECs were visualized by 2′,7′-dichlorofluorescin diacetate (DCFH-DA, Sigma, USA), as previously described [4]. To examine the antioxidant effects of xyloketal B, HUVECs (at a density of 1×10^5^/mL) were seeded in Petri dishes in complete media for 18 h and then serum starved for 6 h. Before loading with DCFH-DA (10 μM) in serum free M199 for 30 min at 37 °C, cells were pretreated with or without SnPP (5, 10 μM) for 30 min and then incubated with xyloketal B (20 μM) for 30 min, followed by incubation with Ang II (10 μM) for 24 h. After washing with HBSS, the fluorescence intensity in Petri dishes was measured at 485 nm excitation and 538 nm emission by a laser confocal scanning microscopy (FV500, Olympus, Japan). Image J 1.37 software was used to estimate the localized mean pixel intensity of the cells for DCF staining (more than 100 cells in each group were recorded). 

### 3.6. Respiratory Burst Assay in Zebrafish

The respiratory burst assay was performed with zebrafish embryos by measuring oxidation of H_2_DCFDA to fluorescent DCF [19]. Embryos in 96-well microtiter plates (Corning Incorporated, USA) were differently pretreated for 30 min with or without 10 μM SnPP and/or with or without 20 μM xyloketal B, respectively, followed by 200 ng/mL PMA (phorbol myristate acetate) incubation. Afterwards, embryos were treated with 1 μg/mL H_2_DCFDA. The intensity of fluorescence was measured every 2.5 min for 150 min by a Multi-Mode Micro-plate Reader (Molecular Device, USA), using excitation and emission filters of 480 ± 10 and 530 ± 10 nm. The intensity of wells containing embryos was normalized to those wells containing culture media only, and the results were expressed as the percentage of fluorescence (%DCF) relative to untreated controls.

### 3.7. RT-PCR Analysis

HUVECs were divided into 6-well plates and treated with 20 μM xyloketal B for 3 h, 6 h, 9 h, 12 h and 24 h. Total RNA was extracted using Trizol (Invitrogen, USA). Zero-point-five μg of total RNA was used to perform the reverse transcription with the 2-step RT-PCR kit (Takara Bio Inc., Japan). The transcribed cDNA was used for polymerase chain reaction (PCR) amplification with specific primers of HO-1 and GAPDH mRNA, and the PCR reaction was carried out as in Table 1 [4,25]. GAPDH (glyceraldehydes 3-phosphate dehydrogenase) served as the loading control. The amplified products were electrophoresed on 2% agarose gels using Gel-Pro Analyzer 6.0 to analyze.

**Table 1:** The polymerase chain reaction (PCR) primers and protocols.

Gene	PCR primer sequences	PCR protocol
HO-1	Forward: GCT CAA CAT CCA GCT CTT TGA GG	95 °C/30 s
(284 bp)	Reverse: GAC AAA GTT CAT GGC CCTGGG A	62 °C/30 s
		72 °C/1 min
GAPDH	Forward: TATCGTGGAAGGACTCATGACC	95 °C/30 s
(625 bp)	Reverse: TACATGGCAACTGTGAGGGG	55 °C/30 s
		72 °C/1 min

### 3.8. Nuclear Protein Extraction

Nuclear protein extracts were prepared according to the manufacturer’s protocol (Active Motif, Carlsbad, CA, USA). Briefly, HUVECs were washed with cold phosphate-buffered saline (PBS)/phosphatase inhibitors, and cells were removed by gently scraping into pre-chilled conical tubes. After centrifugation of the cell suspension at 500 rpm for 5 min, the supernatant was transferred to pre-chilled tubes and stored at −80 °C until ready to use. The cell pellets were resuspended in hypotonic buffer and lysed by adding detergent and then centrifuged at 14,000 *g* for 30 s to obtain a pellet of nuclei. The pelleted nuclei were resuspended in complete lysis buffer and then incubated for 30 min on ice on a rocking platform set at 150 rpm, followed by centrifugation. The supernatant containing the nuclear proteins was used for the following Western blotting analysis. 

### 3.9. Western Blotting Analysis

HUVECs were plated in 60 mm dishes and grown to confluence within 2 to 3 days. Culture medium was then replaced with fresh medium containing 2% FBS, and this was used throughout the xyloketal B experiments. The cells were treated with different concentrations of xyloketal B for a different duration, as indicated in each experiment. Cells were pretreated with or without PI3K/Akt/MAPKs inhibitors 30 min before exposure to xyloketal B. Cellular proteins were harvested, and immunoblots were performed, as previously described [4]. The primary antibodies used were: rat anti-human HO-1 (Abcam, USA), rat anti-human Nrf-2 (Abcam, USA), mouse anti-human Akt, mouse anti-human Erk, mouse anti-human phospho-Akt, mouse anti-human phosphor-Erk (All from Cell Signaling Technology, USA), then the membrane was further incubated for 1 h with appropriate horseradish peroxidase-conjugated secondary antibodies, depending on the primary antibodies, at room temperature. The proteins were detected by enhanced chemiluminescence (ECL, Forevergen, China). The relative densities of target bands were analyzed by Gel-Pro Analyzer 6.0. 

### 3.10. Analysis of Binding Activity of Nrf-2 to Antioxidant Response Element (ARE)

The amount of Nrf-2 available in the nucleus to bind to AREs was determined by a TransAM Nrf-2 Transcription Factor ELISA Kit (Active Motif, USA), according to the manufacturer’s instructions. Briefly, nuclear extracts (2.5 μg) were added to wells containing the immobilized consensus ARE oligonucleotide. A primary antibody against Nrf-2 was added to each well. Then, a secondary antibody conjugated to horseradish peroxidase that binds to the primary (Nrf-2) antibody was added to each well. The signal was detected at 450 nm, and Nrf-2/ARE binding activity was reported as A_450_. 

### 3.11. Statistics

All data are expressed as the mean ± SD. The statistical significance between data from two groups was determined by using a two-tailed Student’s paired *t*-test and a one-way ANOVA with least significant difference (LSD) as *post-hoc* tests performed for comparing multiple quantitative variables. Values of *p* < 0.05 were considered significant.

## 4. Discussion

The present study has revealed that xyloketal B can induce HO-1 and Nrf-2 expression in endothelial cells in a ROS-independent manner. In addition, PI3K and Erk1/2 are involved in induction of HO-1/Nrf-2 by xyloketal B. Most importantly, inhibition of HO-1 by HO-1 inhibitor SnPP significantly abrogates the protective effects of xyloketal B in Ang II-treated endothelial cells. Together with our previous studies, the present data indicates that HO-1 induction may largely contribute to the beneficial actions of xyloketal B in various disease models. 

The present study provides the first direct evidence that xyloketal B can induce HO-1 expression at protein levels under the physiological state. HO-1 is a well-known cytoprotective rate-limiting enzyme in the catabolism of heme into carbon monoxide, biliverdin and iron that acts as an intrinsic potent antioxidant [7]. HO-1 can be induced in endothelial cells and other tissues by a variety of pathophysiological stimuli, including angiotensin II (Ang II) [10]. In the cardiovascular system, Ang II is involved in oxidative stress, vascular tone and vascular remodeling. Given that Ang II plays a critical role in the pathogenesis of cardiovascular diseases, it has been widely used to mimic oxidative damage in cardiovascular diseases. A considerable amount of evidence has suggested a central role of HO-1 induction in preventing Ang II-induced cardiovascular diseases. For example, the induction of HO-1 expression and activity in endothelial cells and aortic smooth muscle cells of heart and kidneys has been reported in response to Ang II-mediated ROS overproduction and oxidative damage, such as cell proliferation, apoptosis and hypertrophy [10]. In addition, genetic and pharmacological inhibition of HO-1 augments Ang II-induced oxidative damage, while enhancing HO-1 expression and activity can significantly attenuate Ang II-induced oxidative damage [26]. Furthermore, the organic nitrate, pentaerythritol tetranitrate, can improve endothelial and vascular dysfunction by inducing HO-1 and then reducing Ang II-mediated superoxide over-production in endothelial cells and vessels [27]. We here found that pretreatment with xyloketal B significantly reduced Ang II-induced cell death, whereas the protection of xyloketal B against Ang-II-induced cell death could be fully abrogated by the HO-1 specific inhibitor, SnPP, suggesting that HO-1 also plays a critical role in the antioxidant properties of xyloketal B. It should be noted that HO-1 is a stress-response protein and can be induced by a variety of stimuli associated with oxidative stress [28]. In fact, some antioxidant compounds induce HO-1 through generation of ROS. In other words, those HO-1 inducers may also act as pro-oxidants under certain conditions, leading to side effects [29]. Thus, it is important to examine whether xyloketal B stimulates HO-1 expression as a pro-oxidant through induction of reactive oxygen species (ROS). In the present study, xyloketal B alone did not stimulate excessive production of ROS, indicating that xyloketal B induces HO-1 expression in a ROS-independent manner. 

The NADPH oxidase family of enzymes is a major source of ROS in the cardiovascular system. Likewise, NADPH oxidase-derived ROS production is a major contributor in the pathophysiological actions of Ang II in cardiovascular diseases [30]. Previously, we have demonstrated that xyloketal B reduces oxHDL-induced cell death through suppression of p47*^phox^*, a major component of NADPH oxidase [4]. Interestingly, it has been reported that HO-1 can directly modulate the activation of p47*^phox^* to inhibit the formation of NADPH assembly [31]. Therefore, we further investigated whether HO-1 induction is also critical for xyloketal B-induced suppression of NADPH oxidase in the zebrafish model. The activity of NADPH oxidase is normally measured by respiratory burst assay *in vitro* using isolated phagocytic cells, such as neutrophils [32]. However, the isolation procedure always affects cell viability. Furthermore, the life span of neutrophils is very short (6–7 h in blood). As a result, the variability of *in vitro* respiratory burst assay is usually high. The zebrafish is widely used as a model system in biomedical research because of its rapid rate of reproduction, the optical clarity of its embryos and the wealth of genetic information [33]. Recently, respiratory burst *in vivo* assay has been established in zebrafish using zebrafish embryos as the sources of phagocytic cells [19]. Respiratory burst assay in zebrafish does not need isolation of phagocytic cells, and this assay has high reliability. Consistent with our *in vitro* experiments, we found in zebrafish embryos that xyloketal B dose-dependently attenuated activation of NADPH oxidase, while the HO-1 inhibitor, SnPP, significantly abrogated the suppressive effects of xyloketal B on NADPH oxidase activation, suggesting that HO-1 plays a critical role in xyloketal B-induced inhibition of NADPH oxidase activation.

The regulation of HO-1 is very complex. In addition to oxidative stress, several transcription factors also regulate HO-1 induction. Among them, nuclear factor erythroid-2-related factor 2 (Nrf-2) is a key mediator for the induction of HO-1 to protect against oxidative stress [10,23]. The HO-1 gene promoter regions contain antioxidant response elements (AREs), and the Nrf-2 family of transcription factors can translocate and bind to ARE in response to oxidative stress, initiating HO-1 gene expression. In the present study, xyloketal B could induce Nrf-2 nuclear translocation in parallel to HO-1 induction, suggesting that Nrf-2 activation may be involved in the induction of HO-1 by xyloketal B. 

Several upstream kinases, such as phosphatidylinositol 3-kinase and mitogen-activated protein kinases, also modulate Nrf-2/HO-1 signaling pathways [10]. The MAPK pathways, including the extracellular signal regulated kinases (ERKs), the cJun-N-terminal kinases (JNKs) and p38-MAPK have been reported to be involved in the regulation of HO-1 activation by different stimuli [10]. The ERK pathway is activated by a large variety of mitogens and by phorbol esters, whereas the c-Jun NH2-terminal kinase (JNK)/stress-activated protein kinase (SAPK) and p38 pathways are stimulated mainly by environmental stress and inflammatory cytokines. Intriguingly, treatment with xyloketal B alone activated the phosphorylation of Akt and Erk1/2, but not P38. Consistently, PI3K or Erk 1/2, but not P38 nor JNK1/2 (data not shown) inhibitors, significantly suppressed the translocation of Nrf-2 in response to xyloketal B, indicating that PI3K/Akt-Erk1/2 MAPKs may be the upstream signal pathways responsible for xyloketal B-induced HO-1/Nrf-2 expression. The PI3K/Akt-dependent pathway is a major regulatory mechanism for eNOS phosphorylation [34]. Xyloketal B has been shown to stimulate NO release from the vascular endothelium through activation of eNOS [4,35]. Interestingly, NO is also a potent HO-1 inducer independent of the redox signaling pathway [36]. Thus, our data suggests that PI3K/Akt may serve as a common pathway responsible for xyloketal B-induced eNOS and HO-1 induction.

The protective mechanisms of HO-1 are only partially understood. HO-1 has been shown to attenuate oxidative stress and maintain mitochondrial integrity through the autophagy process [13]. HO-1 also has strong anti-inflammatory effects. Phagocytic NADPH oxidase of infiltrating proinflammatory monocytes has been shown to induce the inflammatory and oxidative signaling cascade in angiotensin II-induced vascular dysfunction [27]. Therefore, it would be very interesting to explore whether xyloketal B has any role in those two areas in future studies. HO-1 has shown great treatment potential in experimental models of atherosclerotic vascular disease [10,37]. In addition, many therapeutic agents, such as statins, rapamycin, paclitaxel, nitric oxide (NO), aspirin and probucol, have been reported to induce HO-1. Interestingly, HO-1-activating agents are mostly electrophiles [38]. Given that xyloketal B has a unique structure, it would be interesting to test whether xyloketal B is also an electrophile. It also should be noted that many HO-1 inducers also activate Nrf-2. However, long-term Nrf-2 activation leads to prolonged induction of a phase II response, thus having a negative consequence. Therefore, short-term use of Nrf-2/HO-1 inducers should be considered in the clinical setting.

One study limitation is that single concentrations of different pathway inhibitors were used to define the pathways underlying xyloketal B-induced HO-1, which may have issues on specificity. Thus, the interpretation of the present data should be cautious, and further studies using siRNA or similar genetic approaches are warranted to verify the present results. 

## 5. Conclusions

The novel marine compound xyloketal B is a strong natural HO-1 inducer, and xyloketal B-induced HO-1 expression plays a central role in the antioxidative actions of xyloketal B, as evidenced by attenuation of Ang II-induced toxicity in HUVECs and suppression of NADPH oxidase activity in zebrafish embryos. Activation of Nrf-2 and PI3K/Akt and Erk pathways are mainly responsible for xyloketal B-induced HO-1 expression. Xyloketal B is very safe and has a small molecular weight and high liposolubility, which makes it penetrate cell membranes easily. Thus, xyloketal B has a strong potential as a drug candidate to treat oxidative stress-related diseases. In fact, evaluation of xyloketal B as a potential new cardiovascular drug is undergoing in different animal models, and the results yielded are promising. 
